# Primary breast cancer and health related quality of life in Spanish women: The EpiGEICAM case-control study

**DOI:** 10.1038/s41598-020-63637-w

**Published:** 2020-05-08

**Authors:** Nerea Fernández de Larrea-Baz, Beatriz Pérez-Gómez, Ángel Guerrero-Zotano, Ana María Casas, Begoña Bermejo, José Manuel Baena-Cañada, Silvia Antolin, Pedro Sánchez-Rovira, Manuel Ramos Vázquez, José Ángel Garcia-Sáenz, Antonio Antón, Montserrat Muñoz, Ana de Juan, Carlos Jara, José Ignacio Chacón, Angels Arcusa, Miguel Gil-Gil, Encarna Adrover, Amparo Oltra, Joan Brunet, Sonia González, Susana Bezares, Virginia Lope, Miguel Martín, Marina Pollán

**Affiliations:** 1 National Centre for Epidemiology, Instituto de Salud Carlos III (ISCIII), C/Monforte de Lemos, 5, 28029 Madrid, Spain; 2 Consortium for Biomedical Research in Epidemiology and Public Health (CIBERESP), C/Monforte de Lemos, 5, 28029 Madrid, Spain; 3 Medical Oncology Unit, Instituto Valenciano de Oncología, C/Beltrán Báguena, 8, 46009 Valencia, Spain; 4 Medical Oncology Unit, Hospital Virgen del Rocío, Avenida de Manuel Siurot s/n, 41013 Sevilla, Spain; 5 Medical Oncology Unit, Hospital Clínico / INCLIVA, Avenida Blasco Ibáñez, 17, 46010 Valencia, Spain; 6 Medical Oncology Unit, Hospital Universitario Puerta del Mar, Avenida Ana de Viya, 21, 11009 Cádiz, Spain; 7 Biomedical Research and Innovation Institute of Cádiz/Instituto de Investigación e Innovación Biomédica de Cádiz (INiBICA), Cádiz, Spain; 8 Medical Oncology Unit, Complejo Hospitalario Universitario A Coruña, Jubias de Arriba, 84, 15006 A Coruña, Spain; 9 Medical Oncology Unit, Complejo Hospitalario de Jaén, Avenida del Ejército Español, 10, 23007 Jaén, Spain; 10 Medical Oncology Unit, Centro Oncológico de Galicia, Doctor Camilo Veiras, 1, 15009 A Coruña, Spain; 11 Medical Oncology Unit, Hospital Clínico Universitario San Carlos, Profesor Martín Lagos, S/N, 28040 Madrid, Spain; 12 Medical Oncology Unit, Hospital Universitario Miguel Servet, Paseo Isabel La Católica 1-3, 50009 Zaragoza, Spain; 13 Medical Oncology Unit, Hospital Clinic i Provincial, C/Villarroel, 170, 08036 Barcelona, Spain; 14 Translational Genomics and Targeted Therapeutics, Institut d’Investigacions Biomèdiques Pi i Sunyer-IDIBAPS, Barcelona, Spain; 15 Medical Oncology Unit, Hospital Marqués de Valdecilla, Avenida Valdecilla, 25, 39008 Santander, Spain; 16 Medical Oncology Unit, Hospital Universitario Fundación Alcorcón-Universidad Rey Juan Carlos, Calle Budapest, 1, 28922 Alcorcón Madrid, Spain; 17 Medical Oncology Unit, Hospital Virgen de la Salud, Avenida Barber, 30, 45004 Toledo, Spain; 18 Medical Oncology Unit, Consorci Sanitari de Terrassa, Carretera Torrebonica, S/N, 08227 Terrassa, Spain; 19 Medical Oncology Unit, Instituto Catalán de Oncología, Avenida Granvia de l’Hospitalet, 199-203, 08908 L’Hospitalet de Llobregat, Spain; 20 Medical Oncology Unit, Hospital General de Alicante/Complejo Hospitalario Universitario de Albacete, C/Pintor Baeza, 12, 03010 Alicante, Spain; 21 Medical Oncology Unit, Hospital Virgen de los Lirios, Polígono de Caramanchel, S/N, 03804 Alcoy Alicante, Spain; 22 Medical Oncology Unit, Instituto Catalán de Oncología, Avenida de França, S/N, 17007 Girona, Spain; 23 Medical Oncology Unit, Hospital Mutua Terrassa, Plaça Dr. Robert, 5, 08221 Terrassa, Spain; 24 GEICAM Spanish Breast Cancer Group, Avenida de los Pirineos, 7, 28703 San Sebastián de los Reyes Madrid, Spain; 25 Medical Oncology Unit, Instituto de Investigación Sanitaria Gregorio Marañón, C/Doctor Esquerdo, 46, 28007 Madrid, Spain; 26 Complutense University of Madrid, Madrid, Spain; 27 Consortium for Biomedical Research in Oncology (CIBERONC-ISCIII), Madrid, Spain

**Keywords:** Quality of life, Epidemiology, Breast cancer, Cancer epidemiology

## Abstract

This study evaluates the impact of breast cancer (BC) in health related quality of life (HRQL) and in psychological distress (PD) during the initial phases of the disease and looks for contributing factors. A multicentric case-control study, EpiGEICAM, was carried out. Incident BC cases and age- and residence- matched controls were included. Clinical, epidemiological, HRQL (SF-36) and PD information (GHQ-28) was collected. We used multivariable logistic regression models to estimate OR of low HRQL and of PD in cases compared to controls, and to identify factors associated with low HRQL and with PD. Among 896 BC cases and 890 control women, cases had poorer scores than both, the reference population and the control group, in all SF-36 scales. BC women with lower education, younger, active workers, never smokers, those with comorbidities, in stage IV and with surgical treatment had lower physical HRQL; factors associated with low mental HRQL were dissatisfaction with social support, being current smoker and having children. Cases had a fivefold increased odds of PD compared to controls. Managing comorbidities and trying to promote social support, especially in younger and less educated women, could improve well-being of BC patients.

## Introduction

Breast cancer (BC) is the second most frequently diagnosed cancer and the first among women worldwide^1^ and also in Spain^2^. Improvements in survival have contributed to positioning this disease as the most prevalent cancer^3^. Apart from its impact on mortality, BC entails a considerable burden secondary to the associated disability^4^.

Receiving a BC diagnosis can be a very distressing event in a person’s life^5^. Even though distress can diminish with time^6,7^, the news of this diagnosis, frequently associated with the ideas of death and suffering^8^, may by itself affect quality of life. In addition, BC patients may experience physical and psychological symptoms related to the disease and the treatments they receive. As a consequence, different aspects of life, including social relationships or working activities may be significantly impaired. In order to assess this impact, subjective information provided by the patient is highly valuable, and may supplement the clinical information measured by healthcare professionals, resulting in a more precise assessment of patients’ health status.

Health related quality of life (HRQL) is a complex construct that attempts to reflect the impact of health status and health problems in the different facets of people’s life. Although research in this field has increased in the last years, few works have compared HRQL in BC women to that of women in the general population, and inconsistencies have been reported among their results^9,10^. It would be very useful to deepen into the knowledge of the HRQL in newly diagnosed BC patients and, in order to personalise physical and psychological support, to identify patients’ characteristics associated with worse HRQL, and to what extent these associations are also observed in healthy women.

Psychological distress (PD) can be understood as a reaction characterised by emotional instability, which can manifest itself in depression, anxiety or adjustment problems. In BC women, initial PD has been described as a strong predictor of distress along the follow-up^11^. Therefore, the identification of factors associated with higher PD at diagnosis could help to identify, at an early stage, women at risk for future psychological problems.

Our aim in this report is to evaluate the impact of a recent diagnosis of BC on HRQL and on PD in the Spanish EpiGEICAM case-control study, and the factors that modulate these effects. We will explore differences between incident BC cases and control women, as well as in comparison to the reference values for the Spanish female population.

## Results

The scores for all the eight SF-36 scales and the GHQ-28 questionnaire could be calculated in 88.1% of participating BC cases (896/1017) and in 87.5% of controls (890/1017). Some differences were noted between these women and participants excluded due to incomplete response to the SF-36 or the GHQ-28. In both, cases and controls, excluded women were older, had lower education, a different regional distribution and, in those whose scores for specific scales could be computed, worse physical functioning than women with complete SF-36 and GHQ-28. Among cases, in non-respondents there was also a higher proportion of current smokers and higher satisfaction with social support, while there were no differences in clinicopathological characteristics. Finally, scores in the other HRQL scales, apart from the abovementioned physical functioning, were similar between the two groups. In controls, those with incomplete SF-36 or GHQ-28 were more frequently housewives or retired, postmenopausal and showed worse general health, mental health, social functioning and bodily pain (Table 1).

**Table 1:** Comparison of sociodemographic and clinical characteristics between participants with complete and incomplete response to the SF-36 and/or GHQ-28, and between cases and controls.

Variable	Cases					Controls					p-value*†
	Incomplete (N = 121)		Complete (N = 896)		p value*	Incomplete (N = 127)		Complete (N = 890)		p-value*	
	N	%	N	%		N	%	N	%		
**Sociodemographics:**											
**Age (mean and SD)**	121	52.6 (9.7)	896	50.3 (9.5)	0.013	127	53.9 (8.8)	890	49.8 (9.4)	<0.001	0.415
**Ethnic group**					0.461					0.512	0.716
Caucasian	121	100.0%	892	99.6%		127	100.0%	887	99.7%		
Other	0	0.0%	4	0.4%		0	0.0%	3	0.3%		
**Country of birth**					0.448					0.770	0.223
Spain	109	90.1%	826	92.2%		116	91.3%	837	94.0%		
Other	5	4.1%	26	2.9%		3	2.4%	18	2.0%		
Missing	7	5.8%	44	4.9%		8	6.3%	35	3.9%		
**Region of residence**					0.007					0.001	0.875
South	51	42.1%	263	29.4%		44	34.6%	270	30.3%		
East	18	14.9%	210	23.4%		25	19.7%	206	23.1%		
Northeast	14	11.6%	119	13.3%		29	22.8%	103	11.6%		
North	16	13.2%	185	20.6%		12	9.4%	189	21.2%		
Centre	22	18.2%	119	13.3%		17	13.4%	122	13.7%		
**Marital status**					0.162					0.467	0.622
Married/with partner	79	65.3%	669	74.7%		95	74.8%	653	73.4%		
Divorced	14	11.6%	73	8.1%		11	8.7%	67	7.5%		
Single	14	11.6%	97	10.8%		10	7.9%	113	12.7%		
Widow	12	9.9%	55	6.1%		9	7.1%	54	6.1%		
Missing	2	1.7%	2	0.2%		2	1.6%	3	0.3%		
**Cohabiting**					0.253					0.317	0.127
Living with someone else	106	87.6%	807	90.1%		108	85.0%	792	89.0%		
Living alone	14	11.6%	75	8.4%		17	13.4%	94	10.6%		
Missing	1	0.8%	14	1.6%		2	1.6%	4	0.4%		
**Perceived social support**					0.080					0.862	<0.001
Very satisfactory	53	43.8%	408	45.5%		20	15.7%	190	21.3%		
Satisfactory	14	11.6%	226	25.2%		28	22.0%	241	27.1%		
Unsatisfactory	12	9.9%	140	15.6%		28	22.0%	209	23.5%		
Very unsatisfactory	9	7.4%	105	11.7%		29	22.8%	225	25.3%		
Missing	33	27.3%	17	1.9%		22	17.3%	25	2.8%		
**Education**					0.004					<0.001	0.001
No studies or primary school	36	29.8%	184	20.5%		37	29.1%	137	15.4%		
Secondary school	65	53.7%	461	51.5%		62	48.8%	442	49.7%		
University	18	14.9%	246	27.5%		26	20.5%	306	34.4%		
Missing	2	1.7%	5	0.6%		2	1.6%	5	0.6%		
**Socioeconomic level**					0.281					0.663	0.151
Low	25	20.7%	141	15.7%		21	16.5%	128	14.4%		
Intermediate	77	63.6%	623	69.5%		85	66.9%	616	69.2%		
High	11	9.1%	101	11.3%		15	11.8%	126	14.2%		
Missing	8	6.6%	31	3.5%		6	4.7%	20	2.2%		
**Working status**					0.245					<0.001	0.002
Active work (no nightshift)	71	58.7%	477	53.2%		51	40.2%	473	53.1%		
Active work (with nightshift)	1	0.8%	40	4.5%		3	2.4%	80	9.0%		
Retired	10	8.3%	57	6.4%		12	9.4%	51	5.7%		
Housewife	29	24.0%	253	28.2%		51	40.2%	216	24.3%		
Others	7	5.8%	56	6.3%		8	6.3%	60	6.7%		
Missing	3	2.5%	13	1.5%		2	1.6%	10	1.1%		
**Caring for someone**					0.990					0.072	0.645
No	58	47.9%	458	51.1%		76	59.8%	452	50.8%		
Yes, without severe disability	48	39.7%	369	41.2%		40	31.5%	376	42.2%		
Yes, with severe disability	5	4.1%	38	4.2%		3	2.4%	30	3.4%		
Missing	10	8.3%	31	3.5%		8	6.3%	32	3.6%		
**No. comorbidities**					0.121					0.226	0.328
None	60	49.6%	537	58.8%		59	46.5%	490	55.1%		
1	35	28.9%	213	23.8%		41	32.3%	227	25.5%		
2	12	9.9%	92	10.3%		19	15.0%	107	12.0%		
>2	14	11.6%	62	6.9%		8	6.3%	64	7.2%		
Missing	0	0.0%	2	0.2%		0	0.0%	2	0.2%		
**Psychological distress:**											
**GHQ-28 classification**					0.416					0.108	<0.001
No psychological distress	48	39.7%	409	45.6%		80	63.0%	680	76.4%		
Psychological distress	48	39.7%	487	54.4%		35	27.6%	210	23.6%		
Missing	25	20.7%	0	0%		12	9.4%	0	0%		
**Lifestyle:**											
**Smoking**					0.024					0.496	0.234
Never smoker	55	45.5%	389	43.4%		58	45.7%	361	40.6%		
Ex-smoker (>6 months)	19	15.7%	247	27.6%		29	22.8%	235	26.4%		
Current/Ex-smoker<6 months	43	35.5%	259	28.9%		39	30.7%	292	32.8%		
Missing	4	3.3%	1	0.1%		1	0.8%	2	0.2%		
**Adherence to the WCRF/AICR recommendations**‡					0.213					0.194	<0.001
6–9	27	22.3%	158	17.6%		36	28.3%	229	25.7%		
4–5	64	52.9%	514	57.4%		70	55.1%	504	56.6%		
0–3	19	15.7%	191	21.3%		10	7.9%	124	13.9%		
Missing	11	9.1%	33	3.7%		11	8.7%	33	3.7%		
**Adherence to the WCRF/AICR recommendations (mean and SD)**	110	5.1 (1.2)	863	4.9 (1.2)	0.074	116	5.4 (1.1)	857	5.2 (1.1)	0.144	<0.001
**Hormonal factors:**											
**Nulliparous**					0.173					0.794	0.642
No	88	72.7%	701	78.2%		97	76.4%	689	77.4%		
Yes	33	27.3%	195	21.8%		30	23.6%	201	22.6%		
**Menopausal status**					0.176					<0.001	0.288
Pre/perimenopausal	61	50.4%	510	56.9%		41	32.3%	490	55.1%		
Postmenopausal	60	49.6%	386	43.1%		86	67.7%	400	44.9%		
**HRT**					0.407					0.185	0.714
No	90	74.4%	753	84.0%		103	81.1%	760	85.4%		
Yes	15	12.4%	98	10.9%		18	14.2%	92	10.3%		
Missing	16	13.2%	45	5.0%		6	4.7%	38	4.3%		
**Benign breast pathology**					0.439					0.148	0.070
No	98	81.0%	698	77.9%		110	86.6%	724	81.3%		
Yes	23	19.0%	198	22.1%		17	13.4%	166	18.7%		
**BC family history**					0.908					0.118	0.003
None	91	75.2%	668	74.6%		94	74.0%	723	81.2%		
Only SDR	17	14.0%	120	13.4%		16	12.6%	92	10.3%		
First degree relatives	13	10.7%	108	12.1%		17	13.4%	75	8.4%		
**Clinicopathological characteristics:**						N/A	N/A	N/A	N/A	N/A	N/A
**Tumour type**					0.824						
HER2-/HR +	84	69.4%	602	67.2%							
HER2 +	24	19.8%	181	20.2%							
Triple negative	13	10.7%	113	12.6%							
**TNM stage**					0.966						
0	0	0.0%	2	0.2%							
I	41	33.9%	327	36.5%							
II	53	43.8%	384	42.9%							
III	18	14.9%	128	14.3%							
IV	4	3.3%	26	2.9%							
Missing	5	4.1%	29	3.2%							
**Surgery**					0.192						
No surgery	22	18.2%	163	18.2%							
Lumpectomy	53	43.8%	476	53.1%							
Mastectomy	39	32.2%	233	26.0%							
Missing	7	5.8%	24	2.7%							
**Lymphadenectomy**					0.260						
No	80	66.1%	637	71.1%							
Yes	41	33.9%	259	28.9%							
**Radiation therapy**					0.861						
No	92	76.0%	688	76.8%							
Ongoing	13	10.7%	103	11.5%							
Finished	15	12.4%	97	10.8%							
Missing	1	0.8%	8	0.9%							
**Chemotherapy**					0.213						
No	67	55.4%	489	54.6%							
Ongoing	35	28.9%	306	34.2%							
Finished	19	15.7%	97	10.8%							
Missing	0	0.0%	4	0.4%							
**Endocrine therapy**					0.285						
No	94	77.7%	641	71.5%							
Ongoing	26	21.5%	243	27.1%							
Finished	0	0.0%	5	0.6%							
Missing	1	0.8%	7	0.8%							
**HRQL data:**	**N**	**Mean (sd)**	**N**	**Mean (sd)**	**p-value**	**N**	**Mean (sd)**	**N**	**Mean (sd)**	**p-value**	**p-value†**
Physical Functioning	94	60.8 (28.0)	896	65.4 (25.8)	0.147	107	57.0 (30.8)	890	71.5 (28.2)	<0.001	<0.001
Role-Physical	68	34.4 (44.6)	896	30.4 (41.6)	0.662	83	75.2 (36.6)	890	80.5 (34.0)	0.117	<0.001
Bodily Pain	105	56.2 (27.7)	896	58.8 (27.9)	0.437	118	65.1 (27.1)	890	70.0 (24.1)	0.048	<0.001
General Health	78	60.1 (17.2)	896	58.8 (19.6)	0.604	83	63.3 (20.2)	890	69.0 (18.3)	0.013	<0.001
Vitality	88	56.4 (21.6)	896	56.5 (23.1)	0.711	101	57.8 (22.3)	890	60.9 (19.9)	0.134	<0.001
Social Functioning	105	71.5 (25.5)	896	70.3 (27.3)	0.848	120	78.9 (23.1)	890	85.4 (20.6)	0.001	<0.001
Role-Emotional	48	66.7 (41.8)	896	66.7 (43.6)	0.781	70	86.0 (31.6)	890	83.3 (33.1)	0.350	<0.001
Mental Health	87	63.9 (21.3)	896	62.4 (21.3)	0.514	100	63.7 (21.3)	890	70.8 (18.4)	0.001	<0.001

BC: Breast cancer; BMI: Body mass index; DUFSS: Duke-UNC Functional Social Support; HER2: Human Epidermal Growth Factor Receptor 2; HR: Hormone receptors; HRQL: Health related quality of life; HRT: Hormone replacement therapy; N/A: Not applicable; SD: standard deviation; SDR: Second-degree relatives; WCRF/AICR: World Cancer Research Fund/American Institute for Cancer Research.

* p-values estimated without including the missing category. † p-values corresponding to the comparisons between cases and controls with complete questionnaires. ‡ WCRF/AICR recommendations: 1) Be as lean as possible within the normal range of body weight, 2) be physically active as part of everyday life, 3) limit consumption of energy-dense foods and avoid sugary drinks, 4) eat mostly foods of plant origin, 5) limit intake of red meat and avoid processed meat, 6) limit alcoholic drinks, 7) limit consumption of salt and avoid moldy cereals or pulses, 8) aim to meet nutritional needs through diet alone, and 9) mothers to breastfeed. The tenth recommendation, targeted to cancer survivors was not included in the construction of the score of adherence, because it was not applicable to the recently diagnosed population included in our study. Thus, the score ranges from 0 to 9.

The main characteristics of BC cases and controls included in the study are presented in Table 1. BC cases and controls had a similar age (mean: 50 years), more than 90% were born in Spain, more than 70% were married or with a partner, 20% were nulliparous, around 55% of them had no comorbidities, 41–43% were non-smokers and 45% were caring for someone. On the other hand, BC cases reported more social support, had lower education, were more often retired or housewives, had lower adherence to WCRF/AICR recommendations and a higher frequency of BC family history (25% vs 19%).

With respect to clinical characteristics of the cases, 67% had hormone receptor positive and HER2 negative tumours, and 80% were in stages 0-II. At the time of answering the questionnaire, 79% had received surgery, and 40% were receiving radiation therapy or chemotherapy (Table 1).

### Psychometric properties of the questionnaires

SF-36’s internal consistency was good for the whole instrument and per scale. Two scales, role-physical (RP) and role-emotional (RE), showed the highest ceiling and floor effects; these effects were very different –even opposite- by case status (RP: ceiling/floor_controls_ = 68%/11% *vs*. ceiling/floor_cases_ = 22%/59%; RE: ceiling/floor_controls_ = 76%/10% *vs*. ceiling/floor_cases_ = 59%/26%). Also for social functioning (SF), the ceiling was more evident in controls (SF: ceiling/floor_controls_ = 51%/1% *vs*. ceiling/floor_cases_ = 26%/2%). Internal consistency of the GHQ-28 in our sample was good (Cronbach’s α = 0.913 in cases and 0.922 in controls). Indication of a floor effect was observed, mainly among controls, with 13% of cases and 40% of controls scoring 0 points (Supplementary Table S1).

### Health-related quality of life

**Figure 1:**
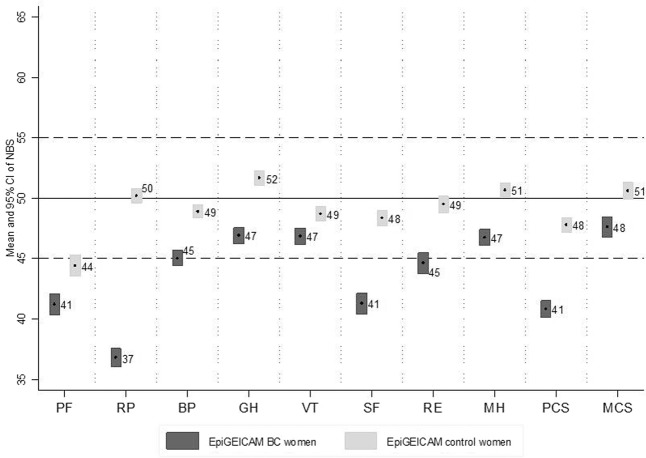
SF-36 means and 95% CI of norm-based scores of EpiGEICAM BC cases and controls. Values under 50 represent a worse perception than the reference population and values over 50, a better perception. All p-values < 0.001. BC:Breast cancer; NBS:Norm-based scores; PF:Physical Functioning; RP:Role-Physical; BP:Bodily Pain; GH:General Health; VT:Vitality; SF:Social Functioning; RE:Role-emotional; MH:Mental Health; PCS:Physical Component Summary; MCS:Mental Component Summary.

**Figure 2:**
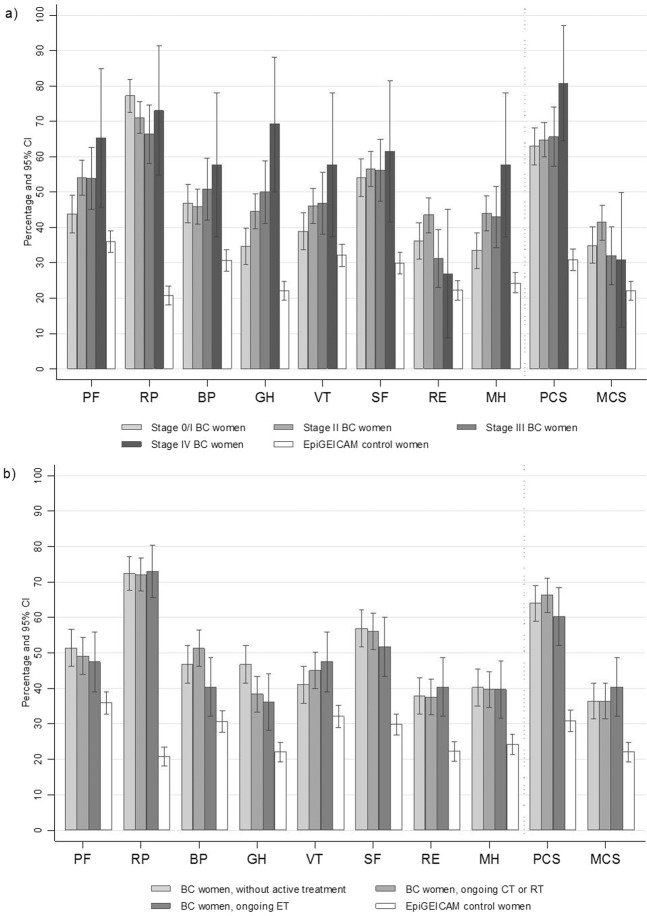
Percentage of women with low scores in the SF-36 scales and component summaries in breast cancer cases stratified by tumour stage (a) and treatment (b), and in control women (EpiGEICAM study). Low scores defined as Norm-based scores five or more points under the Spanish reference population. The category of BC women with ongoing chemotherapy or radiation therapy includes women with or without endocrine therapy; the category of BC women with ongoing endocrine therapy includes those with only endocrine therapy, i.e. not ongoing chemo- or radiation- therapy. BC: Breast cancer; CT: Chemotherapy; ET: Endocrine therapy; RT: Radiation therapy; PF: Physical Functioning; RP: Role-Physical; BP: Bodily Pain; GH: General Health; VT: Vitality; SF: Social Functioning; RE: Role-emotional; MH: Mental Health; PCS: Physical Component Summary; MCS: Mental Component Summary.

In women with BC, mean scores in each of the eight SF-36 scales and in physical and mental component summaries were under 50, showing that these women perceived their health as somewhat poorer than did women in the general population. The scale with the lowest mean was role-physical (36.8 [95%CI:36.0;37.5]) while that with the highest mean was general health (46.9 [95%CI:46.2;47.5]) (Fig. 1). More than 50% of BC cases were classified as reporting low physical functioning, role-physical or social functioning. Focusing on component summaries, 64.7% [95%CI:61.6;67.9] of cases reported low Physical Component Summary (PCS) compared to 30.9% [95%CI:27.9;33.9] of controls, and 37.3% [95%CI:34.1;40.4] of cases reported low Mental Component Summary (MCS) compared to 22.1% [95%CI:19.4;24.9] of controls. Results stratified by tumour stage and by treatment status are shown in Fig. 2.

**Figure 3:**
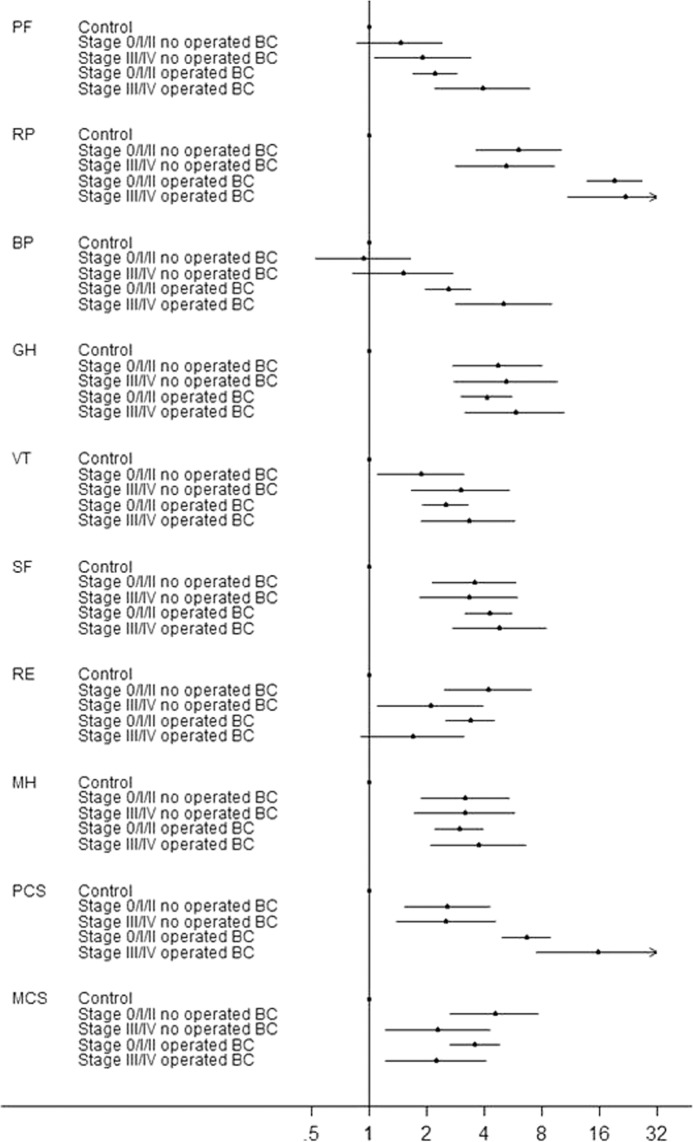
ORs of low scores^a^ in the eight SF-36 scales and the two summary components for breast cancer cases compared to control women in the EpiGEICAM study, by tumour stage and surgical status. ORs adjusted by age, country of birth, region of residence, marital status, perceived social support, education, working status, caregiving, comorbidities, smoking, adherence to the WCRF/AICR recommendations, nulliparity, and family history of breast cancer. Values over 1 indicate higher odds of a low score. ^a^Low scores defined as five or more points under the reference population mean, i.e. ≤45 points. BC: Breast cancer; PF: Physical Functioning; RP: Role-Physical; BP: Bodily Pain; GH: General Health; VT: Vitality; SF: Social Functioning; RE: Role-Emotional; MH: Mental Health; PCS: Physical Component Summary Score; MCS: Mental Component Summary Score.

These differences were maintained after adjusting for potential confounding factors. BC cases had higher odds of reporting low HRQL than controls in all the SF-36 scales, with ORs ranging from 2.3 [95%CI:1.8;2.9] for physical functioning to 14.0 [95%CI:10.4;18.8] for role-physical (Supplementary Fig. 1). In the analysis that differentiates cases in terms of TNM stage and surgical status, differences with respect to the control group in the bodily pain scale were seen only in operated cases; in physical functioning, role-physical and the PCS differences were higher in cases operated than in not operated. On the other hand, for role-emotional and the MCS, differences with respect to controls were higher in patients in the earliest tumour stages, independent of surgical status. For general health, vitality, social functioning and mental health, all cases showed higher odds of low scores compared to controls, being the magnitude of the ORs similar in the four subgroups (Fig. 3).

### Factors related to HRQL

In women with BC, those older, retired, more educated, and smokers or former smokers had lower odds of low PCS. On the other hand, women that reported any comorbidity, those in stage IV and patients who had received surgery for BC treatment showed increased odds of low PCS (Table 2). These associations were similar among controls except for comorbidities, which showed a stronger association with low physical health in controls (p-interaction = 0.016). With respect to the mental component summary, BC women less satisfied with their social support and current smokers had higher odds of low MCS, while nulliparity was associated with reduced odds of low MCS. Among controls, younger women and those reporting first-degree BC relatives showed higher odds of low mental health (p-interaction: 0.005 and 0.019, respectively). The association with social support was stronger in controls than in BC cases, and smoking status and parity were not associated with mental health in control women. Results for specific SF-36 scales are reported in Supplementary Table S2. The sensitivity analyses including only non-metastatic BC patients produced similar results (data not shown).

**Table 2:** Association of sociodemographic, lifestyle, and clinical factors with low scores^a^ in the SF-36 component summaries and with psychological distress.

		Controls N	Cases N	Health Related Quality of Life						Psychological distress		
				SF-36 Physical Component Summary			SF-36 Mental Component Summary			GHQ-28		
				Controls OR(95% CI)	Cases OR(95% CI)	p-hom	Controls OR(95% CI)	Cases OR(95% CI)	p-hom	Controls OR(95% CI)	Cases OR(95% CI)	p-hom
		753	727	**0.70(0.62;0.79)**	**0.69(0.60;0.78)**	0.824	**0.75(0.66;0.86)**	0.98(0.87;1.11)	**0.005**	0.88(0.77;1.00)	0.93(0.83;1.05)	0.457
**Country of birth**	Spain	737	708	1.00	1.00	0.495	1.00	1.00	0.487	1.00	1.00	0.118
	Others	16	19	1.45(0.46;4.59)	0.84(0.29;2.46)		1.39(0.39;4.97)	0.78(0.26;2.28)		0.55(0.12;2.63)	2.64(0.87;8.07)	
**Marital status**	Married/with partner	549	543	**1.00**	1.00	0.504	1.00	1.00	0.410	1.00	1.00	0.127
	Divorced	59	58	**1.91(1.02;3.59)**	1.05(0.54;2.05)		1.77(0.90;3.46)	1.04(0.56;1.94)		1.73(0.89;3.37)	0.89(0.48;1.65)	
	Single	94	84	0.77(0.37;1.63)	1.06(0.53;2.12)		0.89(0.41;1.91)	1.07(0.54;2.09)		0.64(0.30;1.38)	1.04(0.55;1.98)	
	Widow	51	42	0.99(0.47;2.09)	0.80(0.39;1.67)		0.70(0.25;1.95)	1.45(0.70;3.01)		2.02(0.94;4.32)	0.99(0.48;2.02)	
**Perceived social support**	Very satisfactory	169	345	1.00	1.00	0.647	1.00*	1.00*	0.089	1.00*	1.00*	0.228
	Satisfactory	208	184	0.90(0.54;1.50)	0.75(0.50;1.15)		**2.82(1.39;5.71)**	1.04(0.69;1.56)		1.25(0.67;2.35)	0.90(0.61;1.33)	
	Unsatisfactory	179	117	1.37(0.82;2.31)	0.95(0.58;1.56)		**3.87(1.91;7.85)**	**2.41(1.52;3.84)**		**2.10(1.13;3.88)**	**1.68(1.05;2.70)**	
	Very unsatis-factory	197	81	1.23(0.74;2.02)	0.80(0.45;1.42)		**9.32(4.71;18.45)**	**3.93(2.27;6.80)**		**5.60(3.13;10.02)**	**2.43(1.37;4.30)**	
**Education**	No studies/primary school	102	140	1.00*	1.00*	0.201	1.00	1.00	0.920	1.00	1.00*	0.283
	Secondary school	381	378	0.88(0.50;1.55)	0.62(0.37;1.03)		0.75(0.38;1.46)	0.64(0.39;1.04)		0.94(0.50;1.75)	**0.48(0.30;0.78)**	
	University	270	209	**0.44(0.23;0.83)**	**0.51(0.29;0.92)**		0.82(0.40;1.68)	0.67(0.39;1.16)		0.97(0.49;1.92)	**0.50(0.29;0.87)**	
**Working status**	Active work without nightshifts	411	400	1.00	**1.00**	0.263	1.00	1.00	0.845	1.00	**1.00**	0.191
	Active work with nightshifts	75	35	1.26(0.71;2.25)	1.37(0.54;3.46)		0.81(0.41;1.59)	1.18(0.55;2.50)		0.86(0.44;1.68)	**2.44(1.06;5.62)**	
	Retired	43	46	1.26(0.53;2.98)	**0.38(0.17;0.85)**		0.89(0.31;2.58)	0.78(0.35;1.76)		0.49(0.18;1.36)	0.90(0.41;1.99)	
	Housewife	172	198	1.07(0.67;1.69)	0.65(0.41;1.04)		0.90(0.53;1.54)	0.78(0.50;1.21)		0.89(0.53;1.48)	0.71(0.46;1.09)	
	Others	52	48	0.92(0.45;1.85)	0.96(0.47;1.98)		1.33(0.67;2.66)	0.94(0.48;1.84)		1.03(0.50;2.12)	0.95(0.50;1.82)	
**Caring for someone**	No	393	388	1.00	1.00	0.812	1.00	1.00	0.200	1.00	1.00	0.745
	Yes, without severe disability	337	309	0.87(0.59;1.28)	0.98(0.67;1.45)		0.86(0.56;1.33)	0.79(0.55;1.16)		1.10(0.73;1.65)	0.94(0.65;1.35)	
	Yes, with severe disability	23	30	2.17(0.83;5.72)	1.71(0.69;4.25)		1.99(0.72;5.50)	0.59(0.24;1.42)		2.41(0.91;6.38)	1.61(0.68;3.82)	
**Comor bidities**	None	426	447	1.00*	1.00*	**0.016**	1.00	**1.00**	**0.036**	**1.00**	1.00	0.135
	1	185	168	**2.24(1.47;3.40)**	**1.73(1.10;2.73)**		0.81(0.49;1.32)	0.85(0.56;1.29)		0.77(0.48;1.25)	1.26(0.83;1.91)	
	2	88	69	**3.16(1.82;5.46)**	1.47(0.80;2.73)		1.37(0.75;2.51)	**0.42(0.21;0.81)**		1.46(0.82;2.62)	0.90(0.50;1.62)	
	>2	54	43	**8.91(4.46;17.78)**	1.84(0.85;3.99)		1.24(0.57;2.67)	1.66(0.80;3.46)		**2.07(1.03;4.16)**	1.68(0.79;3.57)	
**Smoking**	Never smoker	293	308	1.00	**1.00**	0.132	1.00	1.00	0.403	1.00	1.00	0.166
	Former smoker (>6 months)	207	200	0.91(0.58;1.43)	**0.60(0.39;0.93)**		0.70(0.42;1.15)	1.10(0.72;1.66)		0.86(0.53;1.37)	1.13(0.76;1.70)	
	Current smoker/Former<6 months	253	219	1.15(0.76;1.73)	**0.63(0.40;0.98)**		1.20(0.77;1.88)	1.50(0.99;2.27)		0.80(0.51;1.25)	1.45(0.96;2.19)	
**Adherence to WCRF/AICR recommend-ations**	[6–9]	202	137	1.00	1.00	0.819	1.00	1.00	0.911	1.00*	1.00	0.658
	[4–5]	439	428	0.91(0.61;1.36)	1.02(0.65;1.60)		1.15(0.73;1.83)	1.00(0.65;1.53)		1.48(0.93;2.33)	1.20(0.79;1.83)	
	[0–3]	112	162	0.96(0.55;1.67)	1.22(0.70;2.13)		1.15(0.61;2.16)	1.03(0.61;1.75)		**2.14(1.17;3.91)**	1.43(0.85;2.41)	
**Nulliparous**	No	585	561	1.00	1.00	0.948	1.00	**1.00**	0.225	1.00	**1.00**	0.057
	Yes	168	166	0.61(0.33;1.12)	0.62(0.37;1.07)		0.91(0.48;1.72)	**0.55(0.32;0.94)**		1.04(0.57;1.90)	**0.49(0.30;0.82)**	
**BC family history**	None	615	543	**1.00**	1.00	0.417	**1.00**	1.00	**0.019**	1.00	1.00	0.138
	Only SDR	80	99	0.64(0.35;1.17)	0.84(0.51;1.38)		1.00(0.54;1.85)	1.02(0.63;1.65)		0.86(0.47;1.58)	0.92(0.57;1.47)	
	First degree relatives	58	85	**2.02(1.10;3.69)**	1.32(0.77;2.27)		**2.05(1.06;3.95)**	0.62(0.36;1.06)		1.63(0.86;3.08)	0.75(0.46;1.24)	
**Tumour type**	HER2-/HR +	N/A	493	N/A	1.00	N/A	N/A	1.00	N/A	N/A	1.00	N/A
	HER2 +		145		0.95(0.61;1.47)			0.84(0.55;1.28)			0.67(0.45;1.01)	
	Triple negative		89		0.86(0.50;1.48)			0.83(0.50;1.39)			0.69(0.42;1.14)	
**TNM stage**	0/I	N/A	258	N/A	**1.00**	N/A	N/A	1.00	N/A	N/A	1.00*	N/A
	II		337		1.19(0.79;1.79)			1.22(0.82;1.79)			1.41(0.96;2.06)	
	III		112		1.32(0.74;2.34)			0.71(0.41;1.24)			1.63(0.96;2.76)	
	IV		20		**4.27(1.03;17.82)**			0.46(0.15;1.44)			**5.48(1.61;18.69)**	
**Surgery**	No operated	N/A	138	N/A	**1.00**	N/A	N/A	1.00	N/A	N/A	1.00	N/A
	Operated		589		**3.81(2.34;6.19)**			0.72(0.45;1.14)			1.16(0.73;1.82)	
**Radiation therapy**	No	N/A	573	N/A	1.00	N/A	N/A	1.00	N/A	N/A	1.00	N/A
	Ongoing RT		79		0.83(0.47;1.47)			1.15(0.67;1.99)			1.08(0.63;1.85)	
	Finished RT		75		1.01(0.53;1.93)			0.80(0.44;1.48)			0.89(0.49;1.61)	
**Chemo-therapy**	No	N/A	398	N/A	1.00	N/A	N/A	1.00	N/A	N/A	**1.00**	N/A
	Ongoing CT		253		1.08(0.71;1.66)			1.06(0.71;1.60)			**1.75(1.17;2.61)**	
	Finished CT		76		0.98(0.51;1.91)			1.14(0.62;2.11)			**2.03(1.09;3.76)**	

^a^Low score: scores ≤45 points, i.e. five or more points under the reference population mean, fixed at 50 points. ORs and 95% confidence intervals derived from multivariable logistic regression models, adjusted by all the variables in the table and by region of residence. Data in bold represent statistically significant associations.

*Statistically significant trend. CT: Chemotherapy; HER2: Human epidermal growth factor receptor 2; HR: Hormone Receptor; N/A: Not applicable; RT: Radiation therapy; SDR: Second-degree relatives; p-hom: p homogeneity, estimated from the model including interaction terms between each factor and case/control status.

### Psychological distress

For BC women mean score in the GHQ-28 was 7.4 [95%CI:6.9;7.8], with 54.4% [95%CI:51.1;57.6] being classified as psychologically distressed. In controls, mean score was 3.6 [95%CI:3.2;3.9] and 23.6% [95%CI:20.8;26.4] were classified as psychologically distressed. After adjusting for potential confounding factors, BC cases had a more than fivefold higher odds of PD than controls (OR = 5.7 [95%CI:4.3;7.5]). When taking into account TNM stage and surgical status, this OR was higher in magnitude for the subgroup of patients operated and in stages III/IV (OR = 11.9 [95% CI: 6.4; 22.1]) compared to the other groups (OR = 5.2 [95% CI: 3.1; 8.9] for stage 0-II no operated, OR = 6.9 [95% CI: 3.7; 12.6] for stage III/IV no operated and OR = 5.5 [95% CI: 4.1; 7.4] for stage 0-II operated), although confidence intervals overlapped.

### Factors related to psychological distress

In women with BC, lower satisfaction with social support, lower education, working with nightshifts and having children were related to higher odds of PD. More advanced tumour stage and chemotherapy were also associated with increased odds of PD (Table 2). In controls, the association with perceived social support was stronger than in cases, while no associations were observed with education, working status or parity. Reporting more than two comorbidities and low adherence to the WCRF/AICR recommendations were related to higher odds of PD in control women.

## Discussion

EpiGEICAM BC cases had lower mean scores in all the HRQL domains evaluated by the SF-36, compared with both population reference values and the sample of controls. Role-physical, physical functioning and social functioning were the most affected domains. Psychological distress was very prevalent in BC cases, with more than 50% classified as such by the GHQ-28.

Factors associated with poorer scores in physical health were younger age, lower education, never smoking, active working status, comorbidities, advanced tumour stage and surgery. The association with comorbidities in BC cases was weaker than in controls. This could reflect a response shift among BC women after cancer diagnosis, leading them to give less importance to limitations derived from other chronic diseases. Factors associated with poorer physical health specifically in BC cases were active working and never smoking. Active workers may have a more physically demanding activity than retired women, putting in evidence the limitations caused by the disease or treatments. With respect to smoking, we hypothesise that current and former smokers might attribute some of their physical limitations to consequences of smoking instead of the oncological disease and consequently give higher scores than never smokers in these domains. For mental health, low satisfaction with social support, smoking, and parity were associated with lower scores in BC women, while in controls, a younger age and family history of BC were the factors associated with lower scores. Controls in our study were age-matched to cases, and therefore, younger controls could have had a stronger psychological impact derived from their paired case having received the diagnosis at an earlier age. In contrast, for BC cases, having children seems to have a higher impact on mental health than age, which could reflect that women with children have additional worries related to the impact that her disease can have in their children.

The higher impairment in the physical areas compared to the psychological aspects observed in our results has been reported in other studies^12–15^, and could be related to the time of the HRQL measurement, which took place in the first months after surgery or during radiation therapy or chemotherapy in most participants.

With respect to the high prevalence of PD observed in BC cases in our study, even if it can be overestimated, as has been described with GHQ-28 in people with somatic diseases^16,17^, its magnitude and the differences observed with the control group support the importance of including appropriate screening and attention to psychological problems as part of the integral care for BC patients. Other studies that used the GHQ questionnaire to assess PD in BC women shortly after surgery have also reported elevated prevalences^11,18^. Dissatisfaction with social support, lower education, comorbidities, parity, advanced tumour stage and chemotherapy were the main determinants of PD in our patients. Interestingly, the associations with education and parity were observed only in BC cases, possibly reflecting differences in coping strategies in less educated women and greater concern in patients with children.

Perceived social support was associated in our study with both, PD and the mental component of HRQL. It is worth noting that the percentage of women very satisfied with their social support was much higher among BC cases than among controls, which may reflect a good response from the social networks of these women facing a hard life event. However, there were still some 12% of patients very unsatisfied with their social support, for which interventions aimed at improving it could have a positive impact.

These results should be interpreted in the context of study limitations. First of all, the cross-sectional nature of the design implies that the temporal sequence cannot be stablished for some variables, and we cannot know whether a factor, such as social support is a cause and/or a consequence of the poor HRQL or the PD. However, perceived social support has been found to predict HRQL during follow-up in other studies, giving support to an interpretation of our results in terms of perceived social support preceding HRQL^19,20^. Secondly, even though women participation was high (82%), the sample was not randomly selected and therefore it cannot be considered representative of all the BC patients. In addition, women that did not complete the SF-36 or the GHQ questionnaires were not included in the analyses, leaving women older, less educated and with poorer scores in certain HRQL scales under-represented in our results. To mitigate these limitations we have provided a detailed description of the characteristics of women included in our study in order to facilitate the assessment of external validity. An additional limitation to the external validity is that, by design, women older than 70 years were not invited to participate in our study. Thirdly, although we were able to adjust by several variables, residual confounding could still affect our estimates due to unmeasured variables (e.g. concomitant stressful life events) or to insufficient discriminative capacity of the variables included (e.g. comorbidities were self-reported through an open question). Also, the generic nature of the SF-36 may limit the possibility of evidencing certain disease- or treatment- specific adverse events or consequences. Nevertheless, this instrument has allowed us detecting differences with respect to the reference population and to the group of controls. On the other hand, given that we showed a comprehensive description of SF-36’s results, including the eight individual scales and the two component summaries, and that we did not perform multiple testing adjustments, probability of finding some spurious statistically significant associations could not be ruled out. In this regard, consistency with other studies may help to assess the validity of our findings. The differences observed in ceiling and floor effects between cases and controls may have influenced our results, underestimating differences between these groups. Lastly, it should be noticed that controls were mostly selected among in-law relatives, friends and colleagues of BC cases, and therefore, they could be affected by the situation of their paired case. This would contribute to underestimate the differences in HRQL between cases and controls. However, given the small differences observed in HRQL between EpiGEICAM controls and the reference population, with the exception of physical functioning (Fig. 1), we consider that the magnitude of this underestimation would not be very high.

The current work has also some strengths, such as the high sample size, the exhaustiveness of data collection and the high participation rate. Also, we used the SF-36, which has shown good performance in BC patients, both in the literature^21,22^ and in our own sample. The availability of population based reference values for this instrument allows a richer interpretation of the scores obtained, including an approximation to the impact of BC on HRQL. In addition, the recruitment of a control group also contributed to discern which limitations in HRQL could be more specifically derived from BC.

Results from this study contribute to characterise the impact of a recent breast cancer diagnosis in women’ quality of life and the factors associated with impaired HRQL and with PD. This information could aid to advise women newly diagnosed with BC. Clinical and sociodemographic characteristics found to be related to HRQL and PD, such as age, education and parity could be assessed in clinical practice in order to identify women at increased risk of impaired HRQL and mental status, who may need specific interventions to improve them. Controlling comorbidities and reinforcing perceived social support could contribute, in the context of integral health care, to reduce the impact of BC diagnosis on women’ quality of life. This is particularly important in less educated women, who have greater psychological distress.

## Methods

EpiGEICAM is a case-control study carried out in nine Spanish regions. The study methods have been previously described^23,24^. Briefly, staff of the Oncology Departments of 23 public hospitals, members of the Spanish Breast Cancer Group (GEICAM, http://www.geicam.org/), recruited incident BC cases and controls between 2006 and 2011. To be included, women had to be 18–70 years old, able to answer an epidemiological questionnaire, and give written informed consent. For cases, BC had to be histologically confirmed and diagnosed in the last three months before recruitment. Women with previous history of BC were excluded. Controls were mainly selected by each BC case among their in-law relatives, friends, coworkers, and neighbours, and were matched by age (+/−5 years) and place of residence in a 1:1 ratio. All the participants answered an epidemiological questionnaire. Health related quality of life was measured by the SF-36 (version 1), psychological distress by the GHQ-28^25^ and perceived social support by the Duke-UNC scale^26–28^. A more detailed description of the SF-36 and GHQ-28 instruments and the scoring methods applied is provided in the Supplementary Information online.

The study protocol was approved by the Ethics Committees of the 23 hospitals (Fundación Instituto Valenciano de Oncología; Hospital Universitario Virgen del Rocío de Sevilla; Hospital Universitario Puerta del Mar de Cádiz; Hospital Clínico San Carlos de Madrid; Hospital Clinic de Barcelona; Hospital Clínico Universitario de Valencia; Fundación Hospital Alcorcón; Complejo Hospitalario de Toledo; Hospital Mutua Terrassa; Hospital Universitari de Bellvitge; Hospital General Universitario de Alicante; Hospital Virgen de los Lirios de Alcoy; Hospital Universitari de Girona Dr. Josep Trueta; Hospital Universitari Arnau de Vilanova de Lleida; Fundació d’Osona per a la Recerca i l’Educació Sanitáries (FORES), and by the Regional Institutional Review Boards of Burgos and Soria, Aragón-CEICA, Galicia, Cantabria and Jaén). The study was conducted following the principles of the Declaration of Helsinki and subsequent updates, and all the participants gave written informed consent to participate.

### Statistical methods

Psychometric characteristics of the SF-36 and the GHQ-28 were assessed by exploring the pattern of missing items, floor and ceiling effects, and the internal consistency by calculating the Cronbach’s alpha.

As the analyses were restricted to women with information in all the SF-36 scales and in the GHQ-28, a comparison was made between these women and those not included in the analyses due to incomplete SF-36 or GHQ-28, using Chi-squared and Mann-Whitney U tests for categorical and quantitative variables, respectively. We performed a similar descriptive analysis of the included cases and controls.

For each SF-36 scale and component summary score, we defined a dichotomous variable classifying women as having low HRQL when their Norm-Based Score (NBS) was <= 45 points, that is, five or more points lower than the reference population’s mean, fixed at 50 points. To compare the odds of having low HRQL and of PD between BC cases and controls we used multivariable logistic regression models adjusted by age, country of birth, region of residence, marital status, perceived social support, education, working status, caregiving, number of comorbidities, smoking status, nulliparity, BC family history, and adherence to the World Cancer Research Fund/American Institute for Cancer Research (WCRF/AICR) lifestyle recommendations^29^. This last variable was calculated following previously described methodology^30^. We additionally explored the presence of differences in the odds of low HRQL or PD between cases and controls depending on cases’ tumour stage and surgery status. For this purpose we reclassified cases in four groups according to the combination of TNM tumour stage (0-II *vs*. III-IV) and surgical status (operated *vs*. no operated), and fitted models like those mentioned above.

To identify factors associated with low HRQL and with PD, we performed separated analyses for cases and controls. We used multivariable logistic regression analyses to estimate odds ratios (OR) and 95% confidence intervals (95% CI), including the same covariates as mentioned above and, in the analysis of BC cases, also tumour type, TNM stage, and type of treatment received. Lastly, we explored if the observed associations differed between cases and controls in a statistically significant manner. For this purpose, in the multivariable logistic regression models we included interaction terms between each studied factor and case/control status, and tested their statistical significance. To allow tumour factors to be included in these models, controls were arbitrarily assigned to the categories of no surgery, no radiation therapy, no chemotherapy and stage 0/I in the corresponding variables. As a sensitivity analysis, we applied these same models to the subgroup of non-metastatic BC women.

## Supplementary information

Supplementary Information.

## Data Availability

The datasets generated during and/or analysed during the current study are available from the corresponding authors on reasonable request.
