# Supplementary material for: Primary breast cancer and health related quality of life in Spanish women: The EpiGEICAM case-control study
Source: Sci Rep. 2020 May 8;10:7741. doi: 10.1038/s41598-020-63637-w (PMC7211017; doi:10.1038/s41598-020-63637-w)
Supplement: Supplementary file 1 — Supplementary Information. [file 41598_2020_63637_MOESM1_ESM.pdf]

Supplementary Information

Primary breast cancer and health related quality of life in Spanish women: The EpiGEICAM case-control study

Nerea Fernández de Larrea-Baz, Beatriz Pérez-Gómez, Ángel Guerrero-Zotano, Ana María Casas, Begoña Bermejo, José Manuel Baena-Cañada, Silvia Antolin, Pedro Sánchez Rovira, Manuel Ramos Vázquez, José Ángel Garcia-Sáenz, Antonio Antón, Montserrat Muñoz, Ana de Juan, Carlos Jara, José Ignacio Chacón, Angels Arcusa, Miguel Gil-Gil, Encarna Adrover, Amparo Oltra, Joan Brunet, Sonia González, Susana Bezares, Virginia Lope, Miguel Martín, Marina Pollán.

Contents

Supplementary methods. Description of the instruments and scoring criteria used to measure outcomes...2

Supplementary Table S1. Psychometric properties of the SF-36 and GHQ-28 questionnaires in breast cancer cases and controls (EpiGEICAM study) 4

Supplementary Table S2. Association of sociodemographic, lifestyle, and clinical factors with low scores[a] in the SF-36 scales in breast cancer cases and controls in the EpiGEICAM study....................5

Supplementary Figure 1. ORs of low scores in the eight SF-36 scales and the two summary components for breast cancer cases compared to control women (EpiGEICAM study)....................9

Supplementary methods. Description of the instruments and scoring criteria used to measure outcomes

<u>SF-36</u>

The SF-36 is a generic multidimensional HRQL instrument[1,2] that has been translated and validated in several languages, including Spanish[3]. It explores eight domains -physical functioning (PF, 10 items), role-physical (RP, four items), bodily pain (BP, two items), general health (GH, five items), vitality (VT, four items), social functioning (SF, two items), role-emotional (RE, three items), and mental health (MH, five items)- which draw a health profile. In addition, the instrument allows to calculate two summary scores (Physical and Mental Component Summary measures (PCS and MCS)), through specific weighted combinations of the scores in the eight scales. In this case we used the weights recommended for the Spanish population[4]. For each scale, after taking into account missing items in accordance with the SF-36 scoring manual[5], we transformed the scores into a 0-100 scale, and then normalised them according to the Spanish general female population values by age group[6]. To obtain these Norm-Based Scores (NBS), we subtracted with the mean score of female reference population in the corresponding age-group the score obtained from each woman, and then divided the result by the population standard deviation (SD). In order to homogenise the result to the desired mean and SD (i.e. 50 and 10, respectively), it was multiplied by 10 and summed up 50. This way, values higher than 50 represent better health states than their pairs in the general population and scores under 50 represent poorer health states.

<u>GHQ-28</u>

The GHQ-28 is an instrument designed to screen for non-psychotic psychiatric conditions in the general population. It has also been applied to screen cancer patients for psychological distress, showing good sensitivity and specificity[7]. It has been translated into Spanish and validated[8,9]. This instrument has 28 items with four alternative answers. Different processing methods have been reported to classify respondents. We codified as 0 the first two response options in each item ("Better than usual" and "Same as usual") and as 1 the other two ("Worse than usual" and "Much worse than usual"), and calculated the overall score as

the sum of the items, leading to a range of 0-28. According to criteria previously defined[8,9], we classified as psychologically distressed those women scoring more than five points.

References

1. Ware JE, Sherbourne CD. The MOS 36-item short-form health survey (SF-36). I. Conceptual framework and item selection. *Med Care*. 1992;30(6):473-483.

2. McHorney CA, Ware JE, Raczek AE. The MOS 36-Item Short-Form Health Survey (SF-36): II. Psychometric and clinical tests of validity in measuring physical and mental health constructs. *Med Care*. 1993;31(3):247-263.

3. Alonso J, Prieto L, Antó JM. [The Spanish version of the SF-36 Health Survey (the SF-36 health questionnaire): an instrument for measuring clinical results]. *Med Clin (Barc)*. 1995;104(20):771-776.

4. Vilagut G, Valderas JM, Ferrer M, Garin O, López-García E, Alonso J. [Interpretation of SF-36 and SF-12 questionnaires in Spain: physical and mental components]. *Med Clin (Barc)*. 2008;130(19):726-735.

5. Ware JE, Jr., Snow KK, Kosinski M, Gandek B. *SF-36 Health Survey. Manual & Interpretation Guide*. Boston, Massachusetts: The Health Institute, New England Medical Center; 1993.

6. Alonso J, Regidor E, Barrio G, Prieto L, Rodríguez C, de la Fuente L. [Population reference values of the Spanish version of the Health Questionnaire SF-36]. *Med Clin (Barc)*. 1998;111(11):410-416.

7. Vodermaier A, Linden W, Siu C. Screening for Emotional Distress in Cancer Patients: A Systematic Review of Assessment Instruments. *JNCI Journal of the National Cancer Institute*. 2009;101(21):1464-1488. doi:10.1093/jnci/djp336

8. Lobo A, Pérez-Echeverría MJ, Artal J. Validity of the scaled version of the General Health Questionnaire (GHQ-28) in a Spanish population. *Psychol Med*. 1986;16(1):135-140.

9. Lobo A, Ventura T, Marco C. Psychiatric morbidity among residents in a home for the elderly in spain: Prevalence of disorder and validity of screening. *International Journal of Geriatric Psychiatry*. 1990;5(2):83-91. doi:10.1002/gps.930050205

Supplementary Table S1. Psychometric properties of the SF-36 and GHQ-28 questionnaires in breast cancer cases and controls (EpiGEICAM study)

	Controls					Cases				
**Scale / Item**	**N**	**Missing (%)**	**Ceiling effect (%)**	**Floor effect (%)**	**Cronbach's α**	**N**	**Missing (%)**	**Ceiling effect (%)**	**Floor effect (%)**	**Cronbach's α**
**SF-36**										
Physical functioning	997	2.0	14.9	1.1	0.916	990	2.7	6.2	0.6	0.877
Role-physical	973	4.3	68.2	11	0.889	964	5.2	22	59.1	0.928
Bodily pain	1008	0.9	24.5	1.3	0.819	1001	1.6	16.3	3.1	0.875
General health	973	4.3	2.9	0	0.779	974	4.2	0.9	0	0.717
Vitality	991	2.6	2	0.5	0.830	984	3.2	2.9	1.1	0.847
Social functioning	1010	0.7	51.1	0.5	0.813	1001	1.6	26.2	2.3	0.848
Role-emotional	960	5.6	76.4	10.4	0.870	944	7.2	59	26	0.910
Mental health	990	2.7	4.5	0.1	0.859	983	3.3	3.1	0.1	0.872
**GHQ-28**	1005	1.2	0	40.0	0.922	992	2.5	0.1	12.5	0.913

Supplementary Table S2. Association of sociodemographic, lifestyle, and clinical factors with low scores[a] in the SF-36 scales in breast cancer cases and controls in the EpiGEICAM study.

		Physical Functioning		Role-Physical	
		Controls	Cases	Controls	Cases
		OR (95% CI)	OR (95% CI)	OR (95% CI)	OR (95% CI)
**Age (per 5 years)**		**0.82 (0.73; 0.92)**	**0.77 (0.68; 0.87)**	**0.87 (0.76; 1.00)**	0.99 (0.87; 1.13)
**Country of birth**	Spain	1.00	1.00	1.00	1.00
	Other	0.91 (0.29; 2.87)	0.90 (0.32; 2.57)	0.24 (0.03; 2.02)	1.27 (0.42; 3.83)
**Marital status**	Married/with partner	**1.00**	1.00	**1.00†**	1.00†
	Divorced	**1.90 (1.04; 3.46)**	0.74 (0.40; 1.38)	**3.49 (1.81; 6.74)**	0.59 (0.31; 1.15)
	Single	1.33 (0.69; 2.58)	0.78 (0.41; 1.49)	1.19 (0.53; 2.67)	0.97 (0.49; 1.91)
	Widow	0.99 (0.50; 1.93)	0.81 (0.39; 1.69)	1.40 (0.64; 3.05)	0.63 (0.29; 1.37)
**Perceived social support**	Very satisfactory	**1.00**	1.00	**1.00**	1.00
	Satisfactory	1.26 (0.78; 2.02)	1.05 (0.71; 1.56)	0.78 (0.43; 1.41)	0.75 (0.49; 1.16)
	Unsatisfactory	**1.79 (1.10; 2.93)**	0.90 (0.56; 1.44)	1.04 (0.57; 1.90)	0.76 (0.46; 1.26)
	Very unsatisfactory	**1.68 (1.04; 2.70)**	1.46 (0.85; 2.51)	**1.84 (1.06; 3.20)**	0.97 (0.52; 1.81)
**Education**	No studies or primary school	**1.00***	**1.00***	1.00	1.00
	Secondary school	0.90 (0.53; 1.51)	**0.55 (0.34; 0.89)**	0.68 (0.37; 1.26)	0.89 (0.52; 1.51)
	University	**0.50 (0.28; 0.90)**	**0.42 (0.25; 0.74)**	0.59 (0.30; 1.17)	1.03 (0.56; 1.89)
**Working status**	Active work without nightshifts	1.00†	**1.00†**	1.00	1.00
	Active work with nightshifts	1.16 (0.67; 2.02)	1.65 (0.76; 3.59)	1.84 (0.98; 3.46)	1.44 (0.55; 3.75)
	Retired	1.74 (0.82; 3.72)	0.73 (0.33; 1.61)	1.06 (0.42; 2.67)	0.83 (0.35; 1.94)
	Housewife	1.40 (0.91; 2.16)	0.98 (0.63; 1.51)	1.39 (0.82; 2.37)	0.91 (0.56; 1.47)
	Others	0.72 (0.37; 1.42)	**2.53 (1.24; 5.16)**	2.03 (0.99; 4.15)	0.98 (0.48; 2.01)
**Caring for someone**	No	1.00	1.00	1.00	1.00
	Yes, without severe disability	0.71 (0.49; 1.01)	0.77 (0.53; 1.11)	0.75 (0.49; 1.16)	1.18 (0.79; 1.78)
	Yes, with severe disability	1.42 (0.57; 3.54)	1.40 (0.62; 3.18)	1.45 (0.52; 4.04)	1.20 (0.46; 3.13)
**No. comorbidities**	None	**1.00***	1.00	**1.00***	1.00
	1	**1.59 (1.07; 2.35)**	1.04 (0.69; 1.58)	1.58 (0.98; 2.54)	1.56 (0.97; 2.50)
	2	**2.49 (1.49; 4.15)**	1.50 (0.83; 2.71)	**2.14 (1.18; 3.89)**	0.98 (0.52; 1.88)
	>2	**2.34 (1.25; 4.40)**	1.48 (0.69; 3.18)	**4.78 (2.43; 9.38)**	2.30 (0.94; 5.62)
**Smoking**	Never smoker	1.00	1.00	1.00	1.00
	Former smoker	0.81 (0.53; 1.24)	0.70 (0.47; 1.06)	1.20 (0.72; 1.99)	0.78 (0.50; 1.22)
	Current smoker/Former<6 months	1.17 (0.80; 1.72)	0.78 (0.52; 1.17)	1.28 (0.80; 2.05)	1.18 (0.75; 1.87)
**Adherence to the WCRF/AICR recommendations**	[6-9]	1.00	1.00	1.00	1.00
	[4-5]	0.90 (0.62; 1.31)	1.32 (0.86; 2.02)	1.43 (0.90; 2.28)	1.14 (0.70; 1.84)
	[0-3]	0.94 (0.56; 1.58)	1.20 (0.71; 2.02)	1.35 (0.71; 2.58)	0.92 (0.52; 1.63)
**Nulliparous**	No	1.00	1.00	1.00	1.00
	Yes	0.72 (0.41; 1.25)	0.95 (0.57; 1.58)	0.74 (0.38; 1.44)	0.70 (0.41; 1.21)
**BC family history**	None	1.00	1.00	**1.00***	1.00
	Only SDR	1.05 (0.63; 1.78)	0.83 (0.51; 1.33)	1.03 (0.55; 1.93)	0.85 (0.51; 1.42)
	First degree relatives	1.18 (0.66; 2.13)	0.95 (0.57; 1.58)	**2.39 (1.29; 4.44)**	1.02 (0.58; 1.79)
**Tumour type**	HER2-/HR+	NA	1.00	NA	1.00
	HER2+	NA	0.72 (0.48; 1.10)	NA	1.13 (0.71; 1.79)
	Triple negative	NA	1.32 (0.79; 2.19)	NA	0.81 (0.48; 1.38)
**TNM stage**	0/I	NA	**1.00***	NA	1.00
	II	NA	**1.67 (1.14; 2.46)**	NA	0.82 (0.53; 1.27)
	III	NA	**1.89 (1.10; 3.22)**	NA	0.73 (0.41; 1.32)
	IV	NA	**4.29 (1.32; 13.94)**	NA	1.08 (0.34; 3.37)
**Surgery**	No operated	NA	**1.00**	NA	**1.00**
	Operated	NA	**2.17 (1.36; 3.45)**	NA	**3.30 (2.06; 5.29)**
**Radiation therapy**	No	NA	1.00	NA	1
	Ongoing RT	NA	0.65 (0.38; 1.12)	NA	0.72 (0.40; 1.30)
	Finished RT	NA	0.81 (0.44; 1.48)	NA	0.66 (0.35; 1.28)
**Chemotherapy**	No	NA	1.00	NA	1.00
	Ongoing CT	NA	1.09 (0.73; 1.62)	NA	0.95 (0.61; 1.48)
	Finished CT	NA	1.61 (0.86; 3.02)	NA	0.81 (0.41; 1.59)

Supplementary Table S2 (cont.)

		Bodily Pain		General Health	
		Controls	Cases	Controls	Cases
		OR (95% CI)	OR (95% CI)	OR (95% CI)	OR (95% CI)
**Age (per 5 years)**		**0.81 (0.72; 0.91)**	**0.72 (0.63; 0.81)**	**0.58 (0.50; 0.68)**	**0.67 (0.59; 0.76)**
**Country of birth**	Spain	1.00	1.00	1.00	1.00
	Other	0.32 (0.07; 1.56)	0.75 (0.26; 2.13)	0.46 (0.09; 2.22)	0.49 (0.17; 1.44)
**Marital status**	Married/with partner	1.00	**1.00**	1.00	1.00
	Divorced	1.33 (0.70; 2.51)	0.99 (0.53; 1.85)	1.18 (0.57; 2.45)	0.81 (0.42; 1.54)
	Single	1.25 (0.63; 2.46)	0.88 (0.46; 1.70)	0.70 (0.32; 1.53)	1.29 (0.65; 2.56)
	Widow	1.36 (0.67; 2.73)	**0.41 (0.18; 0.91)**	1.04 (0.39; 2.72)	1.69 (0.78; 3.64)
**Perceived social support**	Very satisfactory	**1.00**	1.00	**1.00**	**1.00***
	Satisfactory	1.22 (0.75; 2.00)	1.23 (0.82; 1.83)	1.70 (0.93; 3.11)	1.00 (0.66; 1.51)
	Unsatisfactory	1.22 (0.73; 2.05)	1.11 (0.69; 1.78)	1.51 (0.79; 2.89)	**1.71 (1.06; 2.76)**
	Very unsatisfactory	**1.64 (1.00; 2.69)**	1.05 (0.61; 1.81)	**3.20 (1.76; 5.83)**	**2.50 (1.42; 4.41)**
**Education**	No studies or primary school	1.00	**1.00***	1.00	1.00
	Secondary school	1.30 (0.73; 2.33)	**0.51 (0.32; 0.84)**	0.74 (0.37; 1.48)	0.77 (0.47; 1.27)
	University	0.94 (0.50; 1.76)	**0.38 (0.22; 0.66)**	0.54 (0.25; 1.14)	1.02 (0.58; 1.78)
**Working status**	Active work without nightshifts	1.00	1.00	1.00	1.00
	Active work with nightshifts	0.88 (0.50; 1.57)	0.82 (0.38; 1.77)	0.86 (0.44; 1.69)	1.51 (0.69; 3.28)
	Retired	1.02 (0.45; 2.31)	0.51 (0.22; 1.18)	1.06 (0.36; 3.06)	0.99 (0.42; 2.33)
	Housewife	0.76 (0.48; 1.23)	0.68 (0.44; 1.05)	0.77 (0.44; 1.33)	1.23 (0.79; 1.93)
	Others	1.80 (0.96; 3.39)	1.22 (0.63; 2.38)	1.34 (0.65; 2.76)	1.14 (0.58; 2.24)
**Caring for someone**	No	1.00	1.00	1.00	**1.00**
	Yes, without severe disability	0.95 (0.65; 1.38)	0.81 (0.56; 1.17)	0.82 (0.53; 1.27)	0.76 (0.52; 1.11)
	Yes, with severe disability	2.66 (0.99; 7.14)	0.94 (0.41; 2.18)	0.89 (0.25; 3.17)	**0.28 (0.10; 0.77)**
**No. comorbidities**	None	**1.00*†**	**1.00*†**	**1.00*†**	**1.00*†**
	1	**1.62 (1.08; 2.44)**	**2.27 (1.48; 3.48)**	1.56 (0.96; 2.54)	**1.93 (1.26; 2.97)**
	2	**2.79 (1.64; 4.72)**	1.33 (0.74; 2.41)	**3.67 (1.99; 6.74)**	1.42 (0.75; 2.68)
	>2	**6.88 (3.54; 13.38)**	**3.38 (1.57; 7.27)**	**9.38 (4.49; 19.61)**	**4.16 (1.91; 9.03)**
**Smoking**	Never smoker	1.00	1.00	1.00	1.00
	Former smoker	1.32 (0.86; 2.01)	0.83 (0.55; 1.25)	0.97 (0.59; 1.61)	0.68 (0.45; 1.04)
	Current smoker/Former<6 months	0.86 (0.57; 1.30)	0.68 (0.44; 1.03)	1.04 (0.65; 1.67)	0.88 (0.58; 1.35)
**Adherence to the WCRF/AICR recommendations**	[6-9]	1.00	1.00	1.00	**1.00***
	[4-5]	1.25 (0.84; 1.86)	1.52 (0.99; 2.33)	0.93 (0.58; 1.49)	1.01 (0.65; 1.57)
	[0-3]	1.09 (0.63; 1.89)	1.39 (0.82; 2.36)	1.17 (0.62; 2.19)	**2.03 (1.18; 3.48)**
**Nulliparous**	No	1.00	1.00	1.00	**1.00**
	Yes	0.77 (0.44; 1.36)	0.62 (0.37; 1.04)	0.90 (0.47; 1.72)	**0.48 (0.28; 0.84)**
**BC family history**	None	1.00	1.00	**1.00†**	1.00†
	Only SDR	0.57 (0.32; 1.03)	1.03 (0.64; 1.65)	1.02 (0.54; 1.92)	1.31 (0.8; 2.12)
	First degree relatives	1.56 (0.86; 2.83)	1.57 (0.93; 2.62)	**2.22 (1.12; 4.40)**	0.69 (0.4; 1.18)
**Tumour type**	HER2-/HR+	NA	1.00	NA	1.00
	HER2+	NA	0.79 (0.51; 1.20)	NA	0.8 (0.52; 1.23)
	Triple negative	NA	0.72 (0.42; 1.20)	NA	1.18 (0.7; 1.99)
**TNM stage**	0/I	NA	1.00	NA	**1.00***
	II	NA	0.99 (0.67; 1.45)	NA	1.42 (0.95; 2.11)
	III	NA	1.48 (0.86; 2.55)	NA	1.60 (0.92; 2.78)
	IV	NA	1.50 (0.51; 4.38)	NA	**3.54 (1.12; 11.22)**
**Surgery**	No operated	NA	**1.00**	NA	1.00
	Operated	NA	**3.39 (2.08; 5.53)**	NA	1.08 (0.68; 1.73)
**Radiation therapy**	No	NA	1.00	NA	1.00
	Ongoing RT	NA	1.26 (0.73; 2.16)	NA	0.91 (0.52; 1.62)
	Finished RT	NA	0.59 (0.33; 1.08)	NA	1.10 (0.59; 2.04)
**Chemotherapy**	No	NA	1.00	NA	1.00
	Ongoing CT	NA	1.02 (0.68; 1.52)	NA	0.89 (0.59; 1.35)
	Finished CT	NA	0.96 (0.52; 1.76)	NA	0.78 (0.42; 1.47)

Supplementary Table S2 (cont.)

		Vitality		Social Functioning	
		Controls	Cases	Controls	Cases
		OR (95% CI)	OR (95% CI)	OR (95% CI)	OR (95% CI)
**Age (per 5 years)**		**0.67 (0.59; 0.76)†**	**0.86 (0.77; 0.97)†**	**0.75 (0.66; 0.85)**	**0.81 (0.72; 0.91)**
**Country of birth**	Spain	1.00	1.00	1.00	1.00
	Other	1.05 (0.32; 3.44)	0.87 (0.33; 2.32)	1.46 (0.46; 4.61)	1.96 (0.68; 5.64)
**Marital status**	Married/with partner	1.00	1.00	**1.00†**	1.00†
	Divorced	1.57 (0.83; 2.95)	0.97 (0.53; 1.79)	**2.55 (1.36; 4.81)**	0.72 (0.39; 1.32)
	Single	1.55 (0.75; 3.22)	1.01 (0.54; 1.92)	1.21 (0.62; 2.38)	1.39 (0.74; 2.63)
	Widow	1.99 (0.95; 4.17)	0.87 (0.42; 1.82)	1.93 (0.91; 4.12)	0.78 (0.38; 1.60)
**Perceived social support**	Very satisfactory	**1.00***	**1.00***	**1.00*†**	**1.00*†**
	Satisfactory	**2.27 (1.32; 3.90)**	1.03 (0.70; 1.52)	**2.42 (1.38; 4.24)**	1.11 (0.75; 1.63)
	Unsatisfactory	**2.61 (1.48; 4.60)**	**1.89 (1.20; 2.97)**	**2.75 (1.54; 4.91)**	**2.34 (1.45; 3.77)**
	Very unsatisfactory	**5.51 (3.21; 9.47)**	**2.26 (1.32; 3.85)**	**6.79 (3.88; 11.87)**	**1.94 (1.12; 3.35)**
**Education**	No studies or primary school	1.00	**1.00**	1.00†	**1.00†**
	Secondary school	0.77 (0.43; 1.37)	**0.50 (0.31; 0.80)**	1.37 (0.74; 2.53)	**0.59 (0.37; 0.94)**
	University	0.59 (0.31; 1.11)	**0.57 (0.33; 0.97)**	1.17 (0.60; 2.29)	0.78 (0.45; 1.33)
**Working status**	Active work without nightshifts	1.00	1.00	**1.00†**	1.00†
	Active work with nightshifts	0.87 (0.48; 1.58)	1.43 (0.69; 2.99)	**0.45 (0.23; 0.87)**	1.48 (0.67; 3.25)
	Retired	0.81 (0.32; 2.03)	0.63 (0.28; 1.41)	0.54 (0.19; 1.51)	0.88 (0.41; 1.92)
	Housewife	1.03 (0.65; 1.65)	0.92 (0.60; 1.41)	1.29 (0.80; 2.08)	0.75 (0.49; 1.14)
	Others	0.92 (0.47; 1.78)	1.41 (0.74; 2.69)	1.13 (0.58; 2.20)	0.79 (0.42; 1.51)
**Caring for someone**	No	1.00	1.00	**1.00†**	**1.00†**
	Yes, without severe disability	0.86 (0.58; 1.26)	0.79 (0.55; 1.13)	1.10 (0.75; 1.62)	**0.66 (0.46; 0.95)**
	Yes, with severe disability	1.65 (0.62; 4.42)	1.13 (0.50; 2.55)	**5.55 (2.01; 15.32)**	1.41 (0.60; 3.30)
**No. comorbidities**	None	**1.00**	**1.00**	1.00	1.00
	1	1.24 (0.81; 1.89)	1.16 (0.78; 1.74)	1.06 (0.69; 1.64)	1.28 (0.85; 1.93)
	2	**2.04 (1.18; 3.51)**	0.97 (0.54; 1.75)	1.43 (0.81; 2.53)	1.01 (0.56; 1.80)
	>2	**3.65 (1.86; 7.16)**	**2.33 (1.11; 4.91)**	1.97 (0.97; 4.00)	2.08 (0.98; 4.40)
**Smoking**	Never smoker	1.00	1.00	1.00	1.00
	Former smoker	0.92 (0.59; 1.42)	0.94 (0.63; 1.40)	0.93 (0.59; 1.45)	0.93 (0.63; 1.39)
	Current smoker/Former<6 months	1.05 (0.70; 1.58)	1.04 (0.69; 1.55)	1.22 (0.80; 1.84)	1.11 (0.74; 1.65)
**Adherence to the WCRF/AICR recommendations**	[6-9]	1.00	1.00	1.00	1.00
	[4-5]	0.82 (0.55; 1.23)	1.15 (0.76; 1.75)	1.34 (0.88; 2.05)	1.24 (0.82; 1.88)
	[0-3]	0.86 (0.50; 1.50)	1.50 (0.90; 2.51)	1.17 (0.66; 2.09)	1.54 (0.92; 2.58)
**Nulliparous**	No	**1.00**	1.00	1.00†	**1.00†**
	Yes	**0.42 (0.22; 0.78)**	0.63 (0.38; 1.05)	1.10 (0.62; 1.93)	**0.45 (0.27; 0.75)**
**BC family history**	None	1.00	1.00	**1.00***	1.00
	Only SDR	0.85 (0.49; 1.49)	1.17 (0.74; 1.86)	1.24 (0.73; 2.12)	0.98 (0.61; 1.56)
	First degree relatives	1.13 (0.60; 2.13)	0.92 (0.56; 1.51)	**2.12 (1.12; 4.00)**	1.02 (0.62; 1.68)
**Tumour type**	HER2-/HR+	NA	**1.00**	NA	1.00
	HER2+	NA	**0.61 (0.41; 0.93)**	NA	0.98 (0.65; 1.46)
	Triple negative	NA	0.92 (0.56; 1.51)	NA	0.65 (0.40; 1.08)
**TNM stage**	0/I	NA	1.00	NA	1.00
	II	NA	1.31 (0.90; 1.91)	NA	1.03 (0.71; 1.51)
	III	NA	1.29 (0.77; 2.18)	NA	1.01 (0.60; 1.71)
	IV	NA	2.72 (0.93; 7.91)	NA	0.81 (0.29; 2.29)
**Surgery**	No operated	NA	1.00	NA	1.00
	Operated	NA	1.17 (0.75; 1.83)	NA	1.16 (0.74; 1.82)
**Radiation therapy**	No	NA	1.00	NA	1.00
	Ongoing RT	NA	1.01 (0.60; 1.70)	NA	0.89 (0.52; 1.52)
	Finished RT	NA	0.65 (0.36; 1.17)	NA	0.90 (0.50; 1.62)
**Chemotherapy**	No	NA	1.00	NA	1.00
	Ongoing CT	NA	0.95 (0.64; 1.40)	NA	1.00 (0.68; 1.48)
	Finished CT	NA	1.27 (0.70; 2.30)	NA	1.04 (0.57; 1.91)

Supplementary Table S2 (cont.)

		Role-Emotional		Mental Health	
		Controls	Cases	Controls	Cases
		OR (95% CI)	OR (95% CI)	OR (95% CI)	OR (95% CI)
**Age (per 5 years)**		**0.77 (0.68; 0.88)**†	0.95 (0.85; 1.08)†	**0.76 (0.66; 0.86)**†	0.91 (0.81; 1.03)†
**Country of birth**	Spain	1.00	1.00	1.00	1.00
	Other	0.70 (0.18; 2.72)	1.02 (0.37; 2.85)	1.16 (0.33; 4.11)	1.97 (0.73; 5.30)
**Marital status**	Married/with partner	**1.00**	1.00	1.00	1.00
	Divorced	**2.94 (1.56; 5.54)**	1.08 (0.59; 2.00)	1.76 (0.90; 3.41)	0.75 (0.40; 1.40)
	Single	0.97 (0.46; 2.05)	1.09 (0.57; 2.10)	1.37 (0.65; 2.90)	0.80 (0.42; 1.53)
	Widow	0.67 (0.24; 1.84)	1.19 (0.58; 2.45)	1.75 (0.80; 3.82)	1.40 (0.68; 2.88)
**Perceived social support**	Very satisfactory	**1.00***	**1.00***	**1.00***	**1.00***
	Satisfactory	1.57 (0.85; 2.93)	1.01 (0.68; 1.52)	1.83 (0.97; 3.46)	1.16 (0.78; 1.72)
	Unsatisfactory	**2.38 (1.29; 4.42)**	**1.78 (1.13; 2.82)**	**3.22 (1.71; 6.05)**	**2.90 (1.83; 4.61)**
	Very unsatisfactory	**4.62 (2.56; 8.37)**	**3.01 (1.76; 5.15)**	**7.19 (3.94; 13.14)**	**2.81 (1.65; 4.81)**
**Education**	No studies or primary school	1.00	1.00	1.00	**1.00***
	Secondary school	0.91 (0.47; 1.76)	0.81 (0.50; 1.30)	0.89 (0.49; 1.64)	**0.62 (0.39; 0.99)**
	University	1.06 (0.52; 2.15)	0.84 (0.49; 1.45)	0.82 (0.42; 1.60)	**0.56 (0.33; 0.96)**
**Working status**	Active work without nightshifts	1.00	1.00	1.00	**1.00**
	Active work with nightshifts	0.93 (0.48; 1.78)	0.95 (0.44; 2.03)	0.74 (0.38; 1.47)	1.21 (0.56; 2.58)
	Retired	0.93 (0.33; 2.62)	0.75 (0.34; 1.67)	1.61 (0.66; 3.92)	1.19 (0.55; 2.60)
	Housewife	1.22 (0.72; 2.05)	0.87 (0.56; 1.34)	1.31 (0.80; 2.15)	0.84 (0.54; 1.29)
	Others	1.62 (0.81; 3.23)	0.92 (0.47; 1.79)	1.75 (0.91; 3.39)	1.68 (0.88; 3.23)
**Caring for someone**	No	**1.00**	1.00	1.00†	1.00†
	Yes, without severe disability	0.82 (0.54; 1.26)	0.73 (0.50; 1.05)	0.84 (0.56; 1.26)	0.83 (0.58; 1.20)
	Yes, with severe disability	**2.99 (1.11; 8.09)**	0.89 (0.38; 2.05)	2.44 (0.93; 6.39)	0.43 (0.17; 1.08)
**No. comorbidities**	None	1.00	1.00	1.00	1.00
	1	0.75 (0.46; 1.22)	0.92 (0.61; 1.39)	0.96 (0.61; 1.52)	0.87 (0.58; 1.32)
	2	1.16 (0.63; 2.14)	0.78 (0.42; 1.44)	1.28 (0.71; 2.29)	0.71 (0.39; 1.30)
	>2	1.67 (0.81; 3.46)	1.90 (0.92; 3.92)	1.51 (0.73; 3.11)	1.24 (0.60; 2.57)
**Smoking**	Never smoker	**1.00**	**1.00**	1.00	1.00
	Former smoker	**0.59 (0.36; 0.98)**	1.19 (0.79; 1.80)	0.96 (0.60; 1.54)	1.17 (0.78; 1.76)
	Current smoker/Former<6 m.	1.07 (0.69; 1.65)	**1.52 (1.01; 2.29)**	1.06 (0.68; 1.64)	1.33 (0.88; 2.00)
**Adherence to the WCRF/AICR recommendations**	[6-9]	1.00	1.00	1.00	1.00
	[4-5]	1.27 (0.81; 1.98)	1.09 (0.71; 1.66)	0.87 (0.57; 1.34)	1.13 (0.74; 1.73)
	[0-3]	0.81 (0.42; 1.56)	1.14 (0.68; 1.92)	1.01 (0.56; 1.80)	1.30 (0.77; 2.19)
**Nulliparous**	No	1.00	1.00	1.00	1.00
	Yes	0.87 (0.47; 1.64)	0.62 (0.37; 1.05)	0.71 (0.38; 1.34)	0.82 (0.50; 1.37)
**BC family history**	None	1.00	1.00	1.00	**1.00**
	Only SDR	1.00 (0.55; 1.82)	0.75 (0.46; 1.21)	1.00 (0.56; 1.79)	1.14 (0.72; 1.81)
	First degree relatives	1.41 (0.72; 2.74)	0.65 (0.38; 1.09)	1.14 (0.58; 2.22)	**0.58 (0.34; 0.98)**
**Tumour type**	HER2-/HR+	NA	1.00	NA	1.00
	HER2+	NA	0.72 (0.47; 1.11)	NA	0.99 (0.66; 1.49)
	Triple negative	NA	1.02 (0.62; 1.67)	NA	0.90 (0.54; 1.49)
**TNM stage**	0/I	NA	1.00	NA	1.00
	II	NA	1.34 (0.91; 1.96)	NA	1.33 (0.91; 1.95)
	III	NA	0.72 (0.42; 1.25)	NA	1.13 (0.66; 1.93)
	IV	NA	0.34 (0.10; 1.16)	NA	2.06 (0.72; 5.84)
**Surgery**	No operated	NA	1.00	NA	1.00
	Operated	NA	0.78 (0.49; 1.24)	NA	1.02 (0.65; 1.61)
**Radiation therapy**	No	NA	1.00	NA	1.00
	Ongoing RT	NA	1.25 (0.73; 2.13)	NA	0.98 (0.57; 1.69)
	Finished RT	NA	0.90 (0.50; 1.64)	NA	0.79 (0.44; 1.43)
**Chemotherapy**	No	NA	1.00	NA	1.00
	Ongoing CT	NA	1.17 (0.78; 1.75)	NA	1.14 (0.77; 1.70)
	Finished CT	NA	1.06 (0.58; 1.94)	NA	1.07 (0.59; 1.95)

[a] Low score: scores ≤45 points, i.e. five or more points under the reference population mean, fixed at 50 points. ORs and 95% confidence intervals derived from multivariable logistic regression models adjusted by all the variables in the table (except tumour-related variables in the analysis of the control group) and by region of residence. Estimates for variables not statistically significantly associated with each scale are depicted in gray. Data in bold represent statistically significant associations. * Statistically significant trend. †Statistically significant interaction between the association in cases and controls. CT: Chemotherapy; HER2: Human epidermal growth factor receptor 2; HR: Hormone Receptor; NA: Not applicable; RT: Radiation therapy; SDR: Second-degree relatives.

Supplementary Figure 1

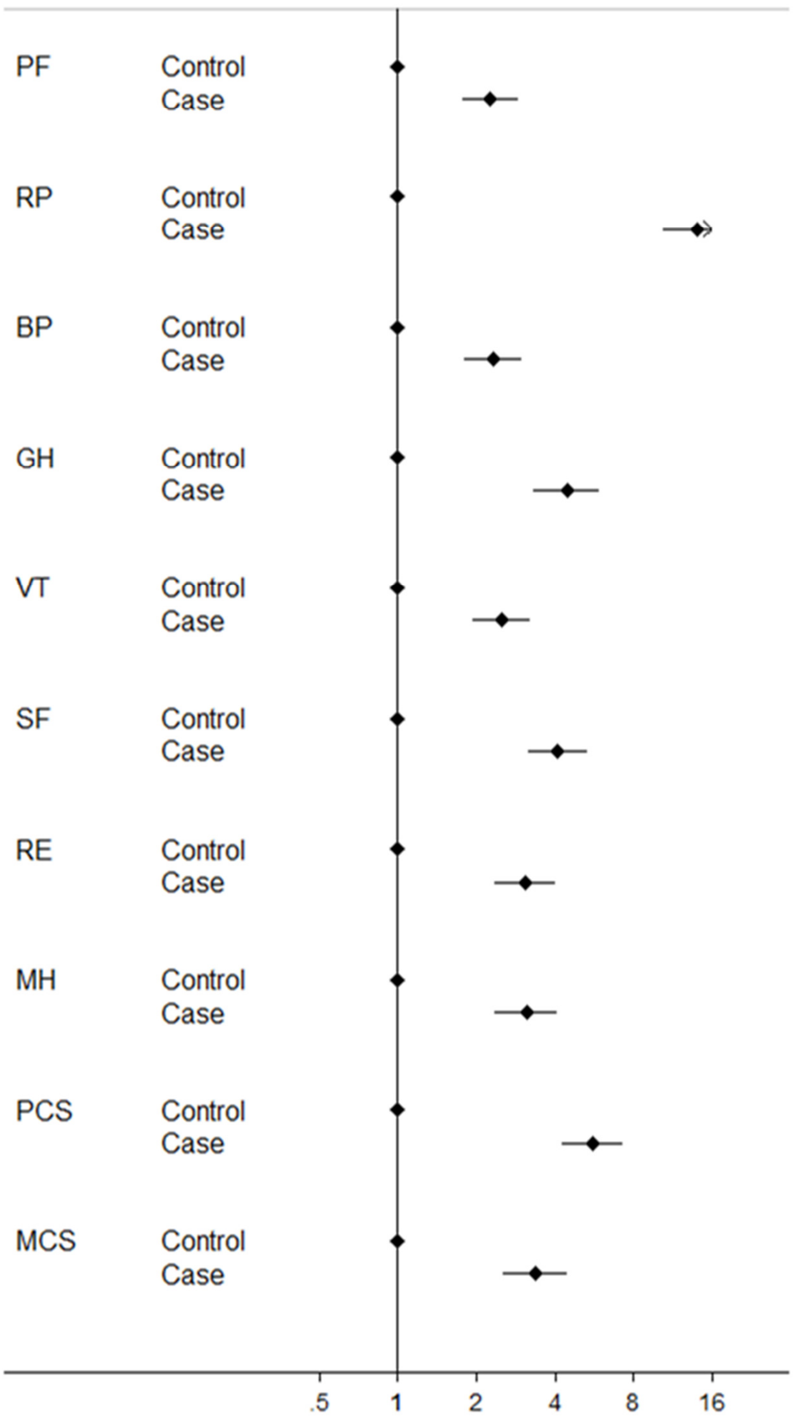

ORs of low scores in the eight SF-36 scales and the two summary components for breast cancer cases compared to control women (EpiGEICAM study).

ORs adjusted by age, country of birth, region of residence, marital status, perceived social support, education, working status, caregiving, comorbidities, smoking, adherence to the WCRF/AICR recommendations, nulliparity, and family history of breast cancer. Values over 1 indicate higher odds of a low score.

PF: Physical Functioning; RP: Role-Physical; BP: Bodily Pain; GH: General Health; VT: Vitality; SF: Social Functioning; RE: Role-emotional; MH: Mental Health; PCS: Physical Component Summary Score; MCS: Mental Component Summary Score.
